# ADAMTS2 and ADAMTS14 can substitute for ADAMTS3 in adults for pro-VEGFC activation and lymphatic homeostasis

**DOI:** 10.1172/jci.insight.151509

**Published:** 2022-04-22

**Authors:** Laura Dupont, Loïc Joannes, Florent Morfoisse, Silvia Blacher, Christine Monseur, Christophe F. Deroanne, Agnès Noël, Alain C.M.A. Colige

**Affiliations:** 1 Laboratory of Tumor and Developmental Biology and; 2 Laboratory of Connective Tissues Biology, GIGA-R, University of Liège, Liège, Belgium.; 3 Institute of Metabolic and Cardiovascular Diseases of Toulouse, INSERM UMR 1048, Toulouse, France.

**Keywords:** Angiogenesis, Vascular Biology, Cardiovascular disease, Growth factors

## Abstract

The capacity of ADAMTS3 to cleave pro-VEGFC into active VEGFC able to bind its receptors and to stimulate lymphangiogenesis has been clearly established during embryonic life. However, this function of ADAMTS3 is unlikely to persist in adulthood because of its restricted expression pattern after birth. Because ADAMTS2 and ADAMTS14 are closely related to ADAMTS3 and are mainly expressed in connective tissues where the lymphatic network extends, we hypothesized that they could substitute for ADAMTS3 during adulthood in mammals allowing proteolytic activation of pro-VEGFC. Here, we demonstrated that ADAMTS2 and ADAMTS14 are able to process pro-VEGFC into active VEGFC as efficiently as ADAMTS3. In vivo, adult mice lacking *Adamts2* developed skin lymphedema due to a reduction of the density and diameter of lymphatic vessels, leading to a decrease of lymphatic functionality, while genetic ablation of *Adamts14* had no impact. In a model of thermal cauterization of cornea, lymphangiogenesis was significantly reduced in *Adamts2-* and *Adamts14*-KO mice and further repressed in *Adamts2/Adamts14* double-KO mice. In summary, we have demonstrated that ADAMTS2 and ADAMTS14 are as efficient as ADAMTS3 in activation of pro-VEGFC and are involved in the homeostasis of the lymphatic vasculature in adulthood, both in physiological and pathological processes.

## Graphical abstract

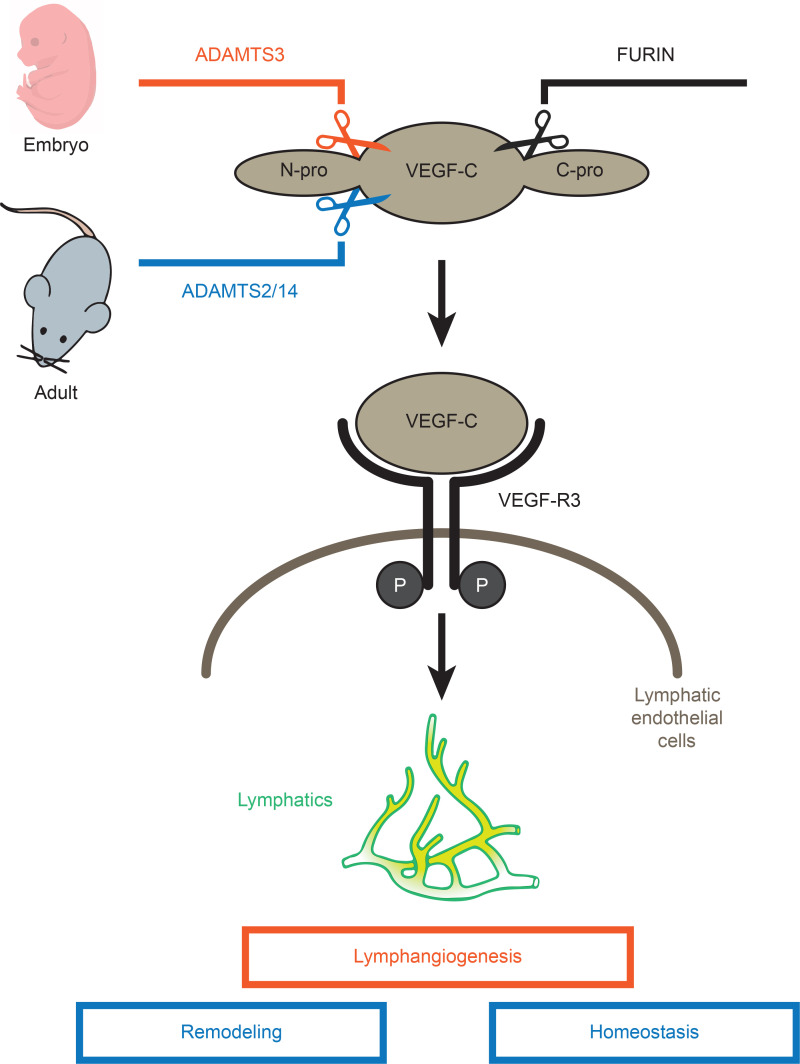

## Introduction

The lymphatic vasculature plays critical homeostatic functions, such as interstitial fluid and macromolecule drainage, local coordination of immunity, and immune cell trafficking (1, 2). The complete absence of lymphatic development leads to embryonic lethality, while lymphatic dysfunctions in adulthood are also implicated in several acquired pathologies, such as secondary lymphedema, psoriasis, and cancer. The most specific lymphatic molecular determinants have been identified through genetic studies of primary lymphatic defects in humans and through the use of transgenic animal models, especially in mice and fish (2, 3). These studies have identified the VEGFC/VEGFR3 axis as a central player in lymphangiogenesis. VEGFC is a ligand of VEGFR3, a receptor mainly expressed by lymphatic endothelial cells (LECs) but also present on blood vessel endothelial cells during angiogenic processes. VEGFD is also a VEGFR3 ligand that is able to stimulate lymphangiogenesis, but its loss in VEGFD-deficient mice and fish does not lead to an obvious phenotype (4). VEGFC and VEGFD are both synthesized as pro-molecules that require the cleavage of the amino- and carboxy terminal domains to acquire the capacity to bind and activate VEGFR3 efficiently (5). The C-propeptides of VEGFC and VEGFD are both cleaved by furin and similar enzymes. However, different processes are implicated in the removal of the N-propeptides. Serine proteases are the processing enzymes for VEGFD, while ADAMTS3 cleaves the N-propeptide of pro-VEGFC, with collagen and calcium-binding EGF domain-containing protein 1 (CCBE1) acting as a cofactor (6, 7). Accordingly, the genetic deletion of *Ccbe1* or *Adamts3* in mouse models leads to similar phenotypes, characterized by a total absence of primary lymphatic network formation, which causes embryonic lethality. In humans, missense hypomorphic mutations in *CCBE1* or *ADAMTS3* (8–10) lead to similar primary lymphedema diseases, Hennekam lymphangiectasia-lymphedema syndrome 1 and 3, respectively. In zebrafish, the absence of *adamts3* does not cause lymphatic defects, as seen in mammals, and only the simultaneous loss of *adamts3* and *adamts14* recapitulates the phenotype seen in the absence of *ccbe1* or *vegfc*. These findings suggest that Adamts3 and Adamts14 can display redundant functions in zebrafish embryo (11).

While the proteolytic activation of pro-VEGFC by ADAMTS3 is now well demonstrated during embryogenesis, it is not likely to occur in adults. Indeed, after birth, ADAMTS3 expression becomes mainly restricted to the central nervous system and the cartilage (12). On the other hand, the proteolytic activation of pro-VEGFC must occur in adulthood for lymphatic homeostasis, as highlighted by models in which the overexpression of pro-VEGFC in adult skin stimulates lymphangiogenesis (13). These observations clearly demonstrate that one or several enzymes can substitute for ADAMTS3 in adults and could therefore be implicated in lymphatic regulation. ADAMTS2 and ADAMTS14 share high sequence homology and an identical domain composition with ADAMTS3 (14). ADAMTS2 is expressed by cells of mesenchymal origin (fibroblasts, smooth muscle cells, adipocytes, etc.), while ADAMTS14 is moderately produced by several cell types, including mesenchymal cells and some immune cells. KO mice have been generated to investigate the in vivo functions of these 3 related ADAMTS proteins. Genetic ablation of *Adamts3* leads to embryonic lethality at 15 days after coitus due to the lack of lymphatic vessel development (7); this has a direct relationship with the capacity of ADAMTS3 to cleave pro-VEGFC into active prolymphangiogenic VEGFC. At birth, *Adamts2*-KO (TS2^–/–^) pups are indistinguishable from their WT littermates. However, within 3 weeks, their skin becomes highly fragile because of the accumulation of incompletely processed procollagen molecules that form abnormal collagen fibrils (15). This clearly illustrates the critical role of ADAMTS2 for the cleavage of the amino-propeptide of fibrillary collagens, an activity at the origin of the name “aminoprocollagen peptidase” given to the subfamily of ADAMTS formed by ADAMTS2, ADAMTS3, and ADAMTS14. Finally, *Adamts14*-KO (TS14^–/–^) mice do not display any obvious morphological phenotype. However, mice deficient for both *Adamts2* and *Adamts14* develop, during aging, skin lesions reminiscent of atopic dermatitis, which are not seen in single-KO mice and suggest dysregulation of the immune system (16). In addition to being able to cleave the amino-propeptide of fibrillar collagens, ADAMTS2, ADAMTS3, and ADAMTS14 can also cleave several common substrates at identical cleavage sites, usually after nonpolar or slightly hydrophobic amino acids, such as Pro, Gly, Ala, or Val (17).

Because ADAMTS2 and ADAMTS14 are mainly expressed in connective tissues where the lymphatic network extends, we reasoned that they could substitute for ADAMTS3 during adulthood in mammals, enabling proteolytic activation of pro-VEGFC at a site (A^111^–A^112^ in humans, A^107^–A^108^ in mice) fully compatible with a cleavage by ADAMTS2 and ADAMTS14. Here, we provide evidence that ADAMTS2 and ADAMTS14 are key actors of pro-VEGFC processing in adulthood.

## Results

### ADAMTS2 and ADAMTS14 control the processing of pro-VEGFC and are thus required to generate its biologically active form.

**Figure 1:**
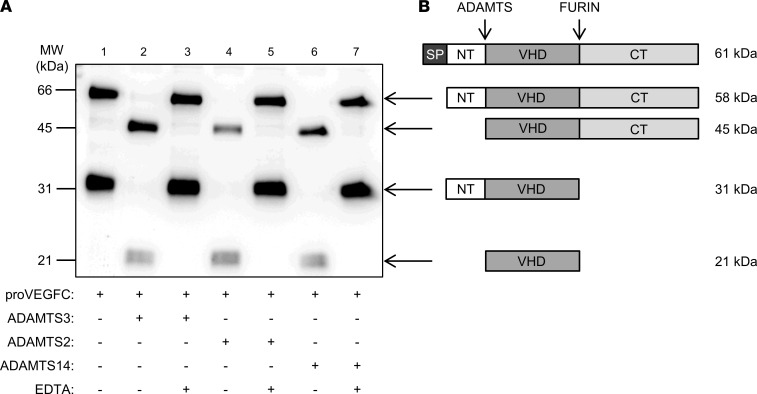
Processing of human pro-VEGFC by ADAMTS3, ADAMTS2, and ADAMTS14 proteases. (**A**) Conditioned medium from HEK293 cells expressing full-length pro-VEGFC was incubated with buffer (as negative control, lane1), ADAMTS3 (as positive control, lane 2), ADAMTS2, or ADAMTS14, in the presence or absence of EDTA used as inhibitor. The electrophoretic pattern of VEGFC was analyzed by Western blotting in reducing conditions. In absence of active enzymes (lane 1, 3, 5, and 7), VEGFC can be detected as a 58 kDa form (full-length pro-VEGFC without signal peptide) and a 31 kDa form generated by C-terminal processing by furin. In the presence of active ADAMTS3 (lane 2), ADAMTS2 (lane 4), and ADAMTS14 (lane 6), the 58 kDa form was totally converted into a 45 kDa polypeptide, whereas the 31 kDa form was processed into the fully mature 21 kDa VEGFC, which is in line with N-terminal processing of VEGFC proteins. (**B**) Schematic illustration of the different VEGFC forms, with their molecular weights provided (SP, signal peptide; NT, N-terminal propeptide; VHD, VEGF homology domain; CT, C-terminal propeptide).

**Figure 2:**
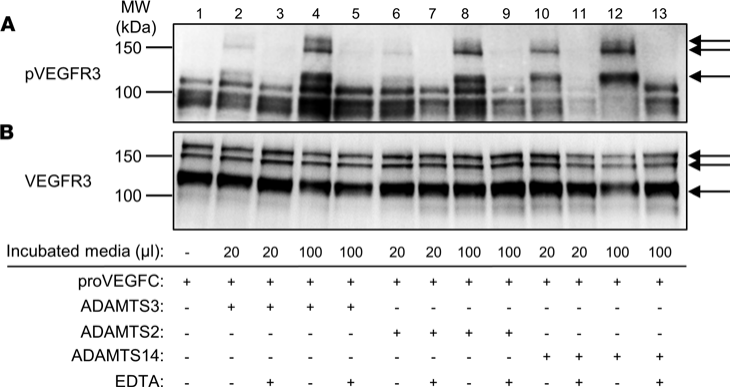
Phosphorylation of VEGFR3 by pro-VEGFC activated or not by ADAMTS3, ADAMTS2, or ADAMTS14. Conditioned medium from HEK293 cells expressing full-length pro-VEGFC was first incubated for 18 hours with buffer alone (lane 1, negative control), ADAMTS3 (as positive control), ADAMTS2, or ADAMTS14, in the presence or absence of EDTA used as inhibitor. These different pretreated media were then added (20 μL or 100 μL) into 1 mL of serum-free EBM-2 on LEC cultures. (**A**) After 5 minutes, cells were lysed, and phosphorylated VEGFR3 (pVEGFR3) was visualized by Western blotting. (**B**) After stripping of the antibodies, the same membrane was then used to visualize total VEGFR3. Treatment of the pro-VEGFC–rich conditioned medium with active ADAMTS3, ADAMTS2, and ADAMTS14 induced the phosphorylation of the 3 bands corresponding to VEGFR3 (arrows) in a dose-dependent manner, while the total amount of VEGFR3 was not affected, demonstrating that processing of pro-VEGFC by ADAMTS2, ADAMTS3, or ADAMTS14 leads to the activation of pro-VEGFC in a similar manner.

In mice, ADAMTS3 is abundant in all connective tissues during embryonic development (12), explaining why, at that stage, it is the main enzyme processing pro-VEGFC into active VEGFC. However, its expression is reported to decrease after birth and to become restricted to brain and cartilage, while ADAMTS2 (mesenchymal cells in general, macrophages, etc.) and ADAMTS14 (fibroblasts, granulocytes, etc.) are more widely expressed (12) (see also The Human Protein Atlas, https://www.proteinatlas.org, and BioGPS, http://biogps.org). We investigated their respective expressions in embryos at 12.5 and 14 days after coitus (dpc) and in the skin of 18 dpc embryos, young pups, and adult mice (Supplemental Figure 1; supplemental material available online with this article; https://doi.org/10.1172/jci.insight.151509DS1). As expected, expression of *Adamts3* was high in 12.5 and 14 dpc embryos, but it was much reduced at 18 dpc and postnatally. In sharp contrast, *Adamts2* and *Adamts14* were produced from day 14 after coitus; their expression remained constant and at a higher level at later stages than the expression of *Adamts3*, suggesting that the activity of *Adamts2* and *Adamts14* could potentially substitute for that of *Adamts3* in their role in lymphangiogenesis during adulthood. To investigate a potential role of ADAMTS2 and ADAMTS14 in lymphangiogenesis, we first evaluated their capacity to activate pro-VEGFC in vitro. The conditioned medium from HEK293 cells overexpressing pro-VEGFC was collected and incubated with recombinant ADAMTS2, ADAMTS14, or ADAMTS3, which was used as a positive control (Supplemental Figure 2). When produced by HEK293 cells, VEGFC is secreted as 2 major forms that correspond to the full-size protein (at 61 kDa) after removal of the signal peptide (at 58 kDa) and to a product (around 31 kDa) resulting from the cleavage of the C-propeptide by pro-protein convertases, such as furin (Figure 1A, lane 1; see Figure 1B for schematic illustration). Upon incubation with ADAMTS3, the cleavage of the N-propeptide was illustrated by the presence of products at 45 kDa and 21 kDa (Figure 1A, lane 2). The same migration pattern was observed after incubation with ADAMTS2 and ADAMTS14, demonstrating that these 2 enzymes can cleave pro-VEGFC (Figure 1A, lanes 4 and 6). EDTA-induced inhibition of ADAMTS3, ADAMTS2, or ADAMTS14 completely abolished pro-VEGFC processing (Figure 1A, lanes 3, 5, and 7). Localization of the cleavage site was determined and demonstrated to occur between Ala^111^ and Ala^112^ for the 3 ADAMTS proteins. Finally, a functional assay has been performed to confirm that this proteolytic processing can indeed activate pro-VEGFC into VEGFC. LECs were treated with pro-VEGFC–enriched medium preincubated with or without ADAMTS2, ADAMTS3, or ADAMTS14. After 5 minutes of treatment, cells were lysed and Western blot analysis was performed using an antibody specific for a phosphorylated form of VEGFR3, which was used as a readout of VEGFR3 activation by the fully processed VEGFC (Figure 2). Receptor phosphorylation was induced when pro-VEGFC was cleaved by ADAMTS3 (compare lane 1 with lanes 2 and 4) but not when ADAMTS3 was inhibited by the presence of EDTA (lanes 3 and 5) (Figure 2A). Most interestingly, the same levels of VEGFR3 phosphorylation were observed when ADAMTS2 (Figure 2A, lanes 6 and 8) or ADAMTS14 (Figure 2A, lanes 10 and 12) were used instead of ADAMTS3. Visualization of total VEGFR3 on the same blot revealed similar patterns for all the samples (Figure 2B), which confirmed that the increased signals observed when pro-VEGFC was incubated with active ADAMTS resulted from increased phosphorylation and not from an increased amount of VEGFR3. Similar results were observed with regard to the phosphorylation of VEGFR2 (Supplemental Figure 3).

### Presence of lymphedema in TS2^–/–^ mice.

**Figure 3:**
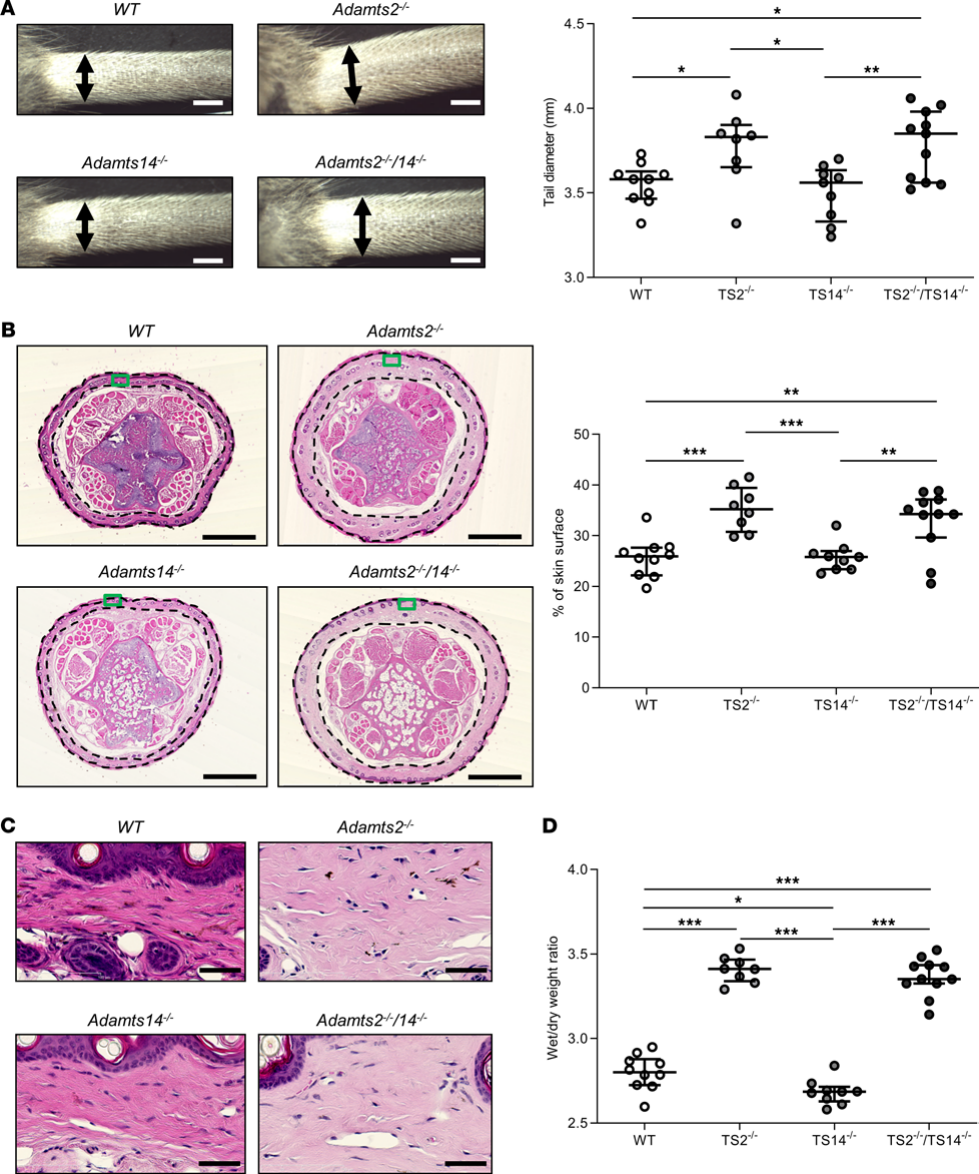
Tail swelling in absence of *Adamts2*. (**A**) Representative images of tails from 8-week-old WT, TS2^–/–^, TS14^–/–^, and TS2^–/–^TS14^–/–^ mice. Base tail diameters were measured (double arrows). Scale bar: 2 mm. As compared with those in WT mice, diameters were increased in TS2^–/–^ and TS2^–/–^TS14^–/–^ mice, suggesting potential lymphedema. (**B**) Hematoxylin and eosin staining of paraffin-embedded sections of tails was performed to further characterize the tissue compartment responsible for the increased diameter. Scale bar: 1 mm. The percentage of surface covered by the dermis (delimited by black dotted lines) was determined (ImageJ software) and was found to be increased in TS2^–/–^ and TS2^–/–^TS14^–/–^ mice. (**C**) Higher-magnification images of boxed regions in **B** show that the dermis is less stained in TS2^–/–^ and TS2^–/–^TS14^–/–^ mice because it is swollen and less dense. Scale bar: 50 μm. (**D**) Tail skins were removed and weighed (wet weight) and then dried for 72 hours in an oven at 60°C before being reweighed (dry weight) to determine the ratios of wet-to-dry weight. An increase of water content was confirmed in TS2^–/–^ and TS2^–/–^TS14^–/–^ mice. Statistical analyses were performed using Kruskal-Wallis test followed by Holm-Šidák post hoc test for multiple comparisons. *P < 0.05; **P < 0.01; ***P < 0.001.

Having clearly demonstrated that ADAMTS2 and ADAMTS14 are as efficient as ADAMTS3 in vitro for activating pro-VEGFC, we investigated if their activity is also functionally important in vivo. We first evaluated signs of potential lymphedema by measuring the diameter of the tail base of adult TS2^–/–^, TS14^–/–^, and TS2^–/–^ and TS14^–/–^ (TS2^–/–^TS14^–/–^) mice. An increased diameter of the tail base was observed for single TS2^–/–^ and double TS2^–/–^TS14^–/–^ mice, as compared with WT mice, but not for single TS14^–/–^ mice (Figure 3A). Edema was associated with histological changes, with swelling of the dermis, as illustrated by an increase of its relative proportion on tissue sections (Figure 3B) and by decrease of its hematoxylin and eosin staining (Figure 3C). Moreover, tail skin samples were also weighed, fresh and after desiccation; this showed that water content was higher in TS2^–/–^ and TS2^–/–^TS14^–/–^ mice (Figure 3D). Similar data were obtained when measuring edema at the level of paws (Supplemental Figure 4).

### Impaired lymphatic network and function in absence of Adamts2 and/or Adamts14 in adult mice.

**Figure 4:**
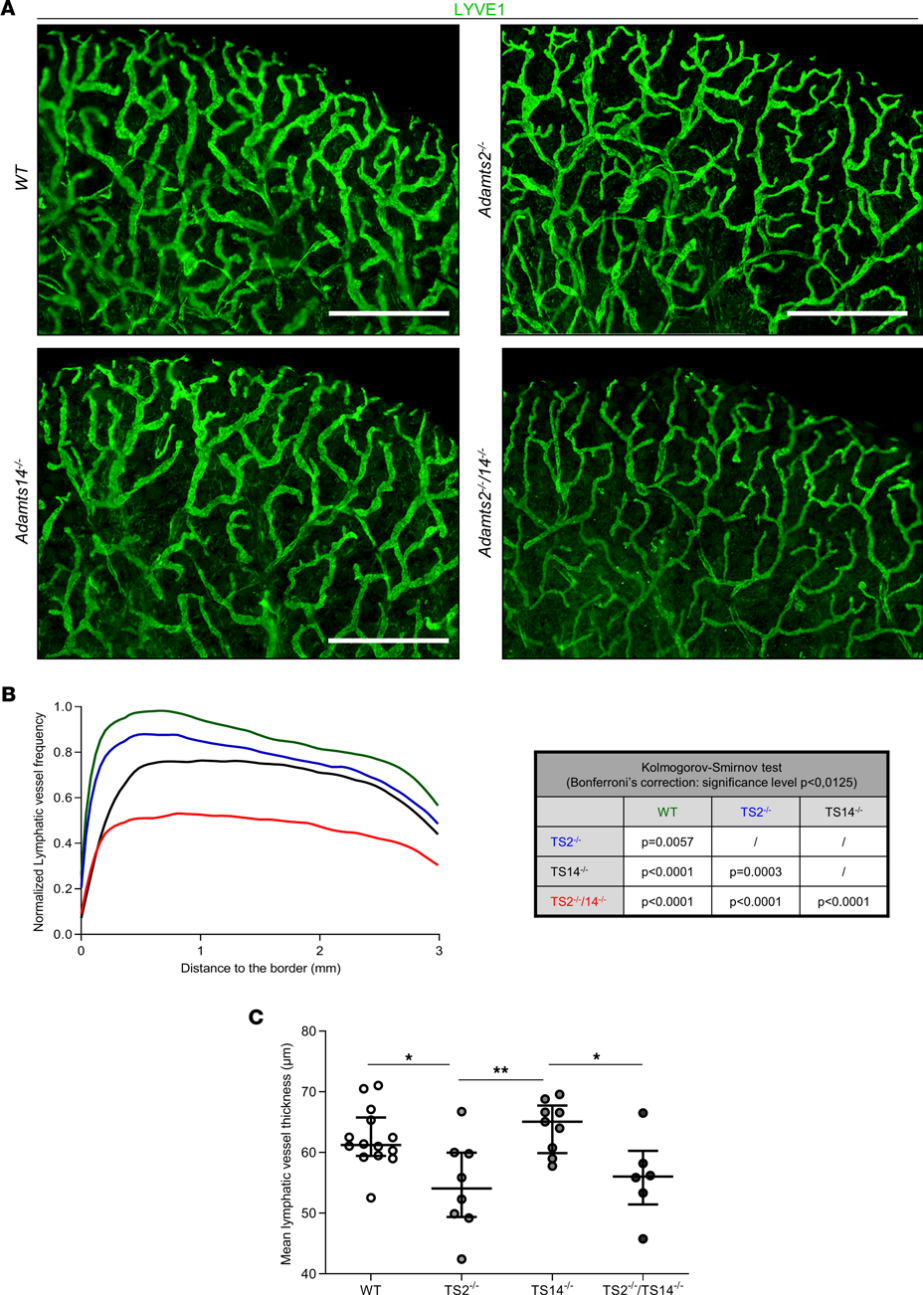
Absence of *Adamts2* and/or *Adamts14* affects the lymphatic network under physiological conditions in adult mice. (**A**) Whole mounts of dorsal ear skin were stained with an antibody specific for LYVE1 for immunofluorescence visualization of lymphatics. Scale bar: 1 mm. (**B**) For computerized quantification, lymphatic vessel frequencies were determined according to the distance from the border of the ear, because the structure and density of the lymphatic network vary according to the distance from the ear edge. The shapes of the distribution curves were found to be statistically different (Kolmogorov-Smirnov test; significance levels were adjusted with Bonferroni’s correction for multicomparison). The most dramatic reduction was observed in TS2^–/–^TS14^–/–^ mice, but it was also found to be reduced in TS2^–/–^ and TS14^–/–^ mice as compared with WT mice. By sharp contrast, no difference was seen regarding blood vessels, showing that these alterations affect lymphatics specifically (Supplemental Figure 5). (**C**) Computerized quantifications of mean diameter of the lymphatic vessels were also performed and demonstrated a smaller diameter in TS2^–/–^ and TS2^–/–^TS14^–/–^ mice. Statistical analyses were performed using Kruskal-Wallis test followed by Holm-Šidák post hoc test for multiple comparisons. *P < 0.05; **P < 0.01.

In order to determine if the lymphatic network is altered in adults lacking *Adamts2* and/or *Adamts14*, whole mounts of dorsal ear skin were stained and evaluated by immunofluorescence. In this model, the structure of the lymphatic network varies according to the distance from the edge of the ear, the vessels being small, highly branched, and dense at the periphery, while being less numerous and larger toward the center. This specific distribution of the ear lymphatic vasculature was taken into account by determining the spatial distribution of normalized lymphatic vessel frequency starting at the ear border. This frequency was significantly reduced in TS2^–/–^ and TS14^–/–^ mice, and it was even more strongly reduced in TS2^–/–^TS14^–/–^ mice The analysis of the curves (Kolmogorov-Smirnov test) indicate that the spatial distributions of the lymphatic vessels were strongly affected in TS2^–/–^TS14^–/–^ mice but also modified in TS2^–/–^ and TS14^–/–^ mice as compared with WT mice (Figure 4, A and B). In sharp contrast, no difference was seen regarding blood vessels, showing that these alterations specifically affect the lymphatic vasculature (Supplemental Figure 5). A reduced diameter of the lymphatics in TS2^–/–^ and TS2^–/–^TS14^–/–^ mice compared with WT and TS14^–/–^ mice was also observed (Figure 4C).

**Figure 5:**
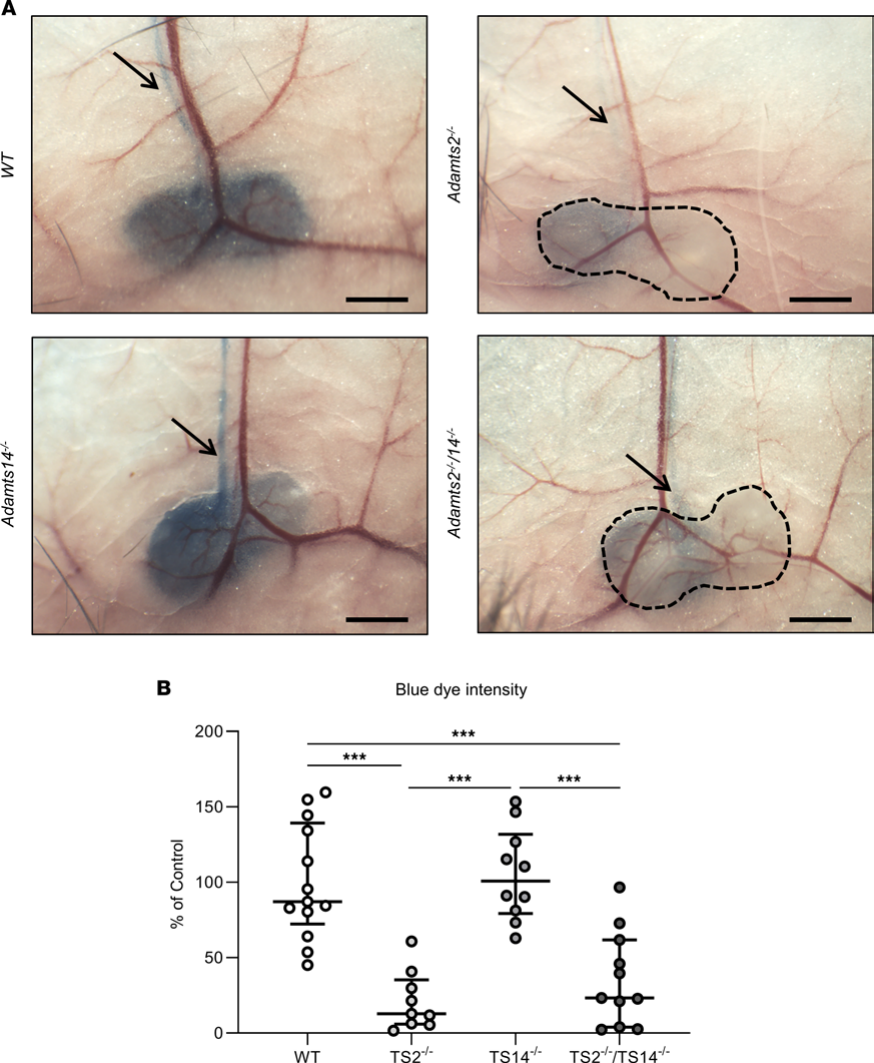
Impaired lymphatic function in absence of *Adamts2*. (**A**) Evans blue dye was injected into the footpad. After 30 minutes, mice were sacrificed and inguinal lymph nodes were visualized. Scale bar: 1 mm. In WT and TS14^–/–^ mice, lymph nodes and the efferent lymphatic vessels (arrows) had a marked blue color. By contrast, they were barely detectable in TS2^–/–^ and TS2^–/–^TS14^–/–^ mice, demonstrating a delayed draining from the site of injection. The images were acquired with a camera-equipped dissection microscope (Optika). (**B**) Computer-assisted quantification of the blue dye intensity in inguinal lymph nodes of WT, TS2^–/–^, TS14^–/–^, and TS2^–/–^TS14^–/–^ mice expressed as a percentage of WT control. Outliers were excluded based on Dixon’s test for extreme values. Statistical analyses were performed using Kruskal-Wallis test followed by Holm-Šidák post hoc test for multiple comparisons. ***P < 0.001.

As a complementary model, to get an insight into potential functional consequences of these alterations, we injected Evans blue dye in the footpads of mice of the 4 genotypes and evaluated its presence in inguinal lymph nodes. Thirty minutes after the injection, lymph nodes and the efferent lymphatic vessels had a marked blue color in WT and TS14^–/–^ mice, whereas they were barely detectable in TS2^–/–^ and TS2^–/–^TS14^–/–^ mice, demonstrating a delayed draining from the site of injection and, therefore, a clear reduction of the functionality of the lymphatic network (Figure 5).

### Involvement of Adamts2 in postnatal lymphatic network homeostasis.

**Figure 6:**
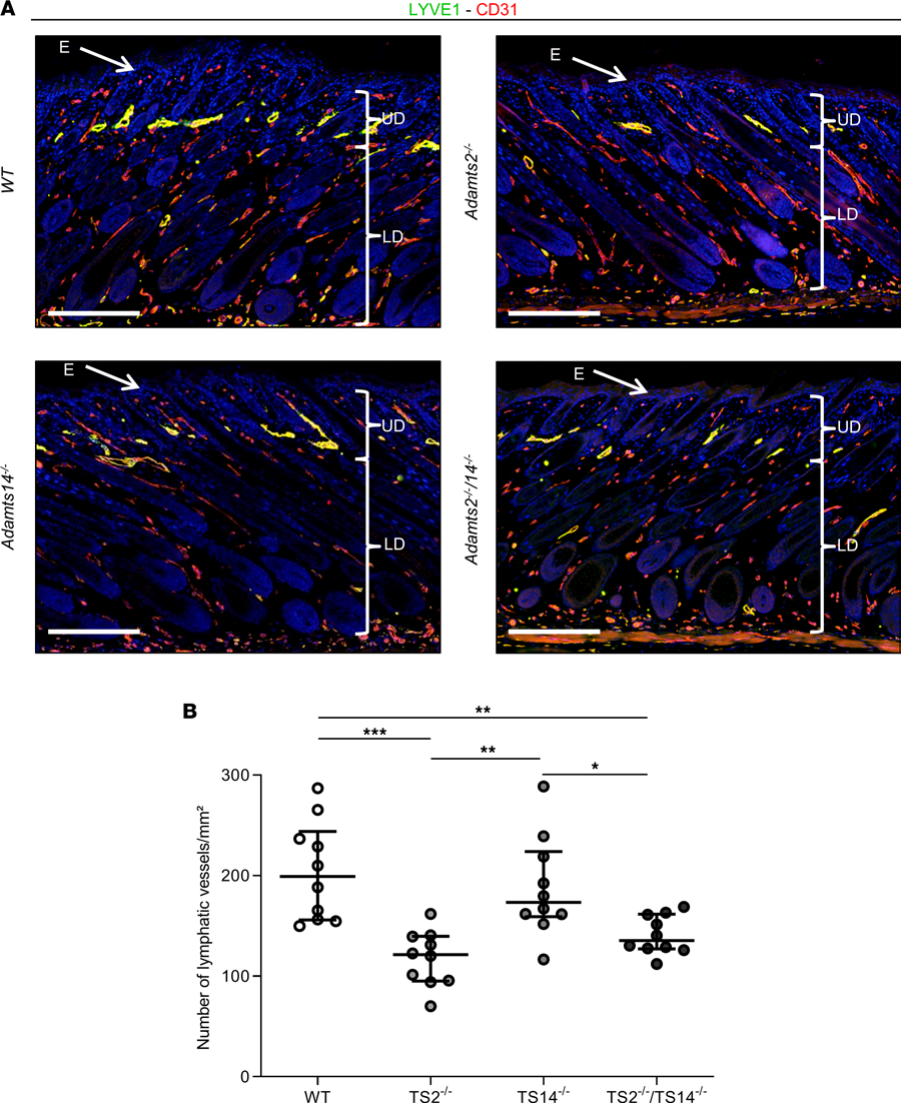
Absence of *Adamts2* affects lymphatic formation in postnatal day 6 mice. Skin sections were stained using antibodies for LYVE1 (green) and CD31 (red). Scale bar: 200 μm. (**A**) Because of coexpression of LYVE1 and CD31, lymphatics appear as yellow structures, mainly in the upper dermis and in close association with the bulge region of the hair follicle (18). LYVE1-positive cells identified in the lower dermis and in the adipose tissue were mainly macrophages (Supplemental Figure 6). (**B**) Computerized quantification of LYVE1 staining in the upper dermis. As compared with WT mice, fewer lymphatics were observed in TS2^–/–^ and TS2^–/–^TS14^–/–^ mice. E, epidermis (white arrows); UD, upper dermis; LD, lower dermis. Statistical analyses were performed using Kruskal-Wallis test followed by Holm-Šidák post hoc test for multiple comparisons. *P < 0.05; **P < 0.01; ***P < 0.001.

In order to evaluate if *Adamts2* and *Adamts14* participate in the postnatal homeostasis of the lymphatic network in vivo, skin samples were collected from 6-day-old pups. In WT mice, LYVE1 staining revealed the presence of lymphatics in the upper dermis in close association with the bulge region of the hair follicle (Figure 6), as expected (18), while the LYVE1-positive cells identified in the lower dermis and in the adipose tissue were mainly macrophages (Supplemental Figure 6). The density of lymphatics was not modified in TS14^–/–^ mice, but markedly reduced in TS2^–/–^ and TS2^–/–^TS14^–/–^ mice (Figure 6), indicating that lymphedema observed in the adulthood most probably results from early postnatal defects.

### ADAMTS2 and ADAMTS14 ablation affects pathological lymphangiogenesis.

**Figure 7:**
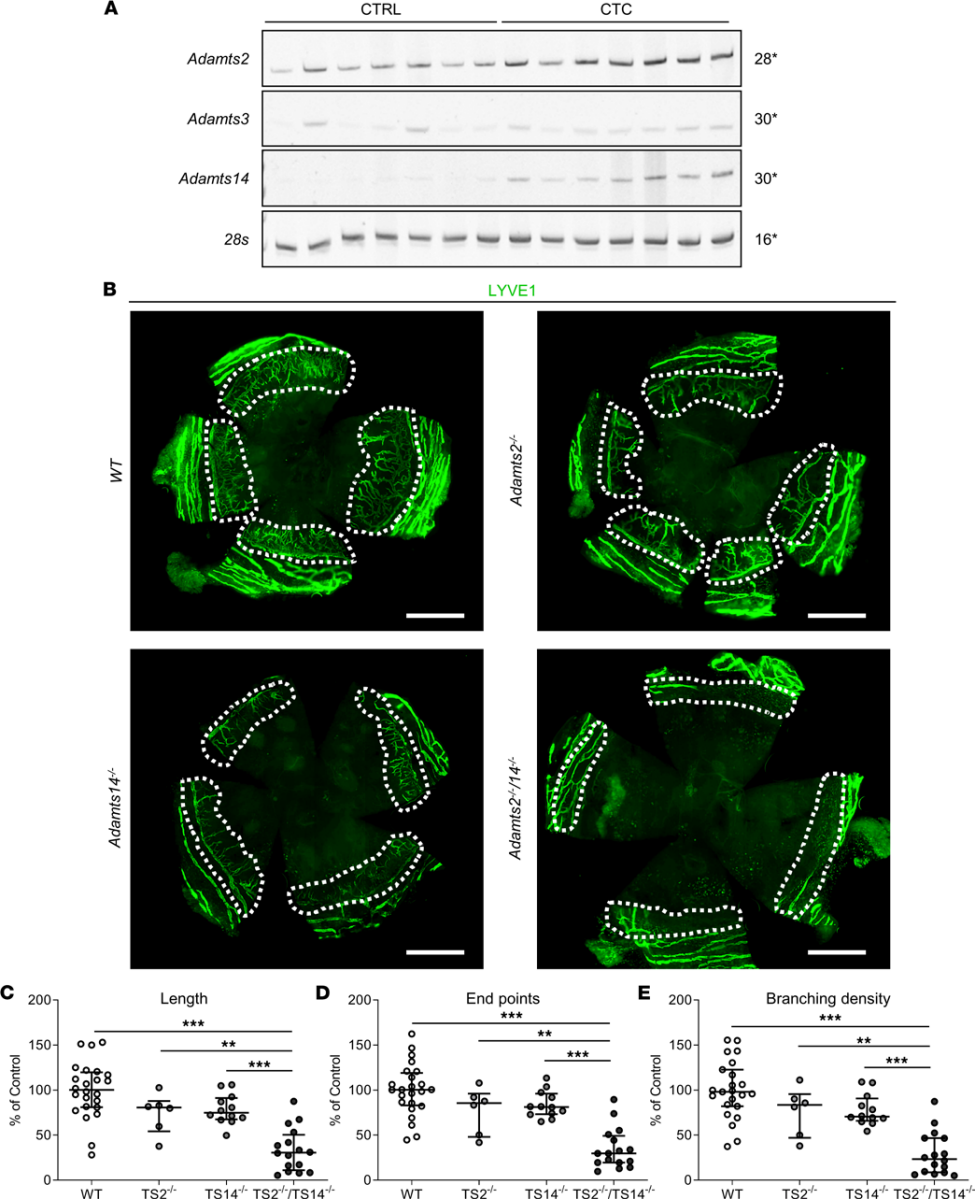
Absence of *Adamts2* or *Adamts14* inhibits inflammatory corneal lymphangiogenesis. (**A**) Expression of *Adamts2* (28 cycles), *Adamts3* (30 cycles), *Adamts14* (30 cycles), and *28s* (as control, 16 cycles) by RT-PCR on corneas collected from uninjured (CTRL) WT mice (*n =* 7) or from WT mice 7 days after corneal thermal cauterization (CTC) (*n =* 7). The expressions of *Adamts2* and *Adamts14*, but not of *Adamts3*, were increased during the healing process of the injured cornea. (**B**) Lymphatic vessel visualization using LYVE1 immunofluorescence on whole-mount corneas 7 days after thermal cauterization in WT, TS2^–/–^, TS14^–/–^, and TS2^–/–^TS14^–/–^ mice. Scale bar: 1 mm. A decrease of lymphatic vessels invading the injured corneas (white dotted lines) were observed in KO mice as compared with WT mice. Computerized quantification of the (**C**) length, (**D**) end point numbers, and (**E**) branching density of lymphatic vessels in corneas 7 days after thermal cauterization in WT, TS2^–/–^, TS14^–/–^, and TS2^–/–^TS14^–/–^ mice expressed as a percentage of WT control (*n =* 23 WT, *n =* 6 TS2^–/–^, *n =* 12 TS14^–/–^, and *n =* 16 TS2^–/–^TS14^–/–^ corneas). Statistical analyses were performed using Kruskal-Wallis test followed by Holm-Šidák post hoc test for multiple comparisons. **P < 0.01; ***P < 0.001.

To investigate the mechanism leading to lymphatic vessel neoformation in adults, we used the model of thermal cauterization-induced corneal lymphangiogenesis, which relies on VEGFC signaling and mimics lymphangiogenesis induced upon inflammatory conditions such as keratitis, chemical burns, and graft rejection (19, 20). Although the cornea is an avascular tissue, it is rapidly colonized after thermal cauterization by lymphatic vessels arising perpendicularly from the limbal vascular arcade. In WT mice, *Adamts2* was expressed in control corneas, and its expression was still increased upon thermal cauterization (Figure 7A). While *Adamts3* remained poorly expressed under physiological and pathological conditions, *Adamts14* was clearly upregulated during the repair process. As compared with WT mice, a drastic reduction of neolymphangiogenesis was observed in TS2^–/–^TS14^–/–^ mice in terms of vessel length, number, and branching (Figure 7, B–E). Interestingly, intermediate values were observed in TS2^–/–^ and TS14^–/–^ mice, suggesting that the 2 enzymes are equally involved in the process. It is worth noting that the effect of ADAMTS deficiency was restricted to the lymphatic system, because the network of corneal blood vessels remained unaffected (Supplemental Figure 7).

## Discussion

The processing of pro-VEGFC into VEGFC is a rate-limiting step for lymphangiogenesis, because it is required to endow VEGFC with its capacity to bind and activate VEGFR3 and VEGFR2. ADAMTS3 is involved in this crucial function during embryonic lymphangiogenesis (6, 7). However, given its restricted expression pattern in adults, it is unlikely that ADAMTS3 still performs this function in adulthood.

ADAMTS3 possesses high similarity with ADAMTS2 and ADAMTS14 in terms of sequence and domain composition. These 3 enzymes also share the capacity to cleave the amino-propeptide of fibrillar collagens but with different efficacy: ADAMTS2 is the most efficient, and ADAMTS3 and ADAMTS14 display only moderate and low activities, respectively (12, 14, 17, 21, 22). By sharp contrast, we have shown here that the 3 enzymes display similar processing activities when pro-VEGFC was used as substrate, showing that it might be a more physiological substrate for ADAMTS14 than fibrillar procollagens and, therefore, that ADAMTS14 activity could be more important for lymphangiogenesis than for collagen maturation. So far, TS2^–/–^ or TS14^–/–^ mice have been used mainly to determine their implications in collagen biology and were never specifically investigated for potential lymphatic defects. Of note, however, TS2^–/–^TS14^–/–^ mice display a peculiar phenotype, consisting of skin lesion formation caused by localized accumulation of inflammatory cells (16), a feature that could result from lymphatic defects because lymphatic vasculature plays a crucial role in resolving inflammation (23).

Potential existence of skin lymphedema was first evidenced by measuring the diameter of the tail base and of paw. Significant differences were found for TS2^–/–^ and TS2^–/–^TS14^–/–^ mice, as compared with WT mice, which were further confirmed on histological tissue sections, by measurements of their skin water content and by the strong reduction of fluid drainage with both the inguinal lymph node and the efferent lymphatic vessel being barely stained after Evans blue dye injection in the footpad. In order to get better insight into the causes leading to reduced drainage, we performed ear skin whole-mount staining in order to get an overview of the lymphatic network. As compared with WT mice, we found a reduction of the density of lymphatic vessels in TS2^–/–^ and TS14^–/–^ mice that was even more pronounced in TS2^–/–^TS14^–/–^mice. This clearly indicates that both enzymes are involved and can partially compensate for each other in single-KO mice. In addition, in absence of *Adamts2* (TS2^–/–^ and TS2^–/–^TS14^–/–^), vessels seem to have a smaller diameter, being shrunk or less swollen, as compared with WT and TS14^–/–^ mice. These data suggest that edema found in TS2^–/–^ and TS2^–/–^TS14^–/–^ mice is caused by decreased functionality of the lymphatic vessels and that their slightly reduced density and branching in TS14^–/–^ mice is not sufficient to induce edema in physiological conditions. The skin of 6-day-old pups was also analyzed in order to determine if alteration of the lymphatic network takes place progressively in the adulthood or very early after delivery. For technical reasons related to the size and the fragility of samples, these analyses were performed on histological cross-sections of back skin. Skin lymphatics do not have specific orientations, and, as a result, their surfaces on histological sections vary strongly according to their local orientation. Therefore, we quantified the number of LYVE1-stained structures per mm² rather than the surface they covered, so that lymphatics studied in cross-sections or longitudinal sections would have the same values. Only the absence of *Adamts2* had an affect on lymphatic density, which could be attributed to the residual presence of *Adamts3* a few days after birth and to the fact that *Adamts2* is expressed much more than *Adamts14* around birth.

Having shown the effect of *Adamts2* on lymphatics in physiological conditions, we then used a model of acute neolymphangiogenesis occurring after injury. In the eye, lymphatic vessels are found in the conjunctiva and the limbus, in which lymphatic vasculature is a ring-shaped network with very small extensions directed toward the central avascular cornea (24). However, upon inflammatory stimuli, blood and lymphatic vessels rapidly extend from the limbus toward the cornea. The major advantage of the corneal neovascularization assay is that any vessel that grows into the corneal stroma is newly formed and can easily be detected, allowing the determination of several relevant parameters, such as vessel length, invasion distance, or vascular density (24). In the absence of *Adamts2* or *Adamts14*, a significant reduction of cornea invasion was observed, with lymphatics being shorter, less numerous, and less branched, as compared with WT mice. Remarkably, these alterations are more obvious in TS2^–/–^TS14^–/–^ mice, demonstrating that both enzymes actively participate in lymphangiogenesis during tissue inflammation and repair. Of note, angiogenesis was not affected, arguing for the involvement of the VEGFC/VEGFR3 pathway, as VEGFC is required for lymphangiogenesis but outcompeted by VEGFA for stimulating angiogenesis.

It was known that ADAMTS3 is required for embryonic lymphangiogenesis because of its capacity to process pro-VEGFC into active VEGFC. Here, we have demonstrated that ADAMTS2 and ADAMTS14 are as efficient as ADAMTS3 for processing pro-VEGFC into active VEGFC and that their absence in mouse models in vivo leads to alterations of the lymphatic network in adulthood.

These data open research avenues that are beyond the scope of the present study. As an example, it is not clear yet whether CCBE1 promotes the activity of ADAMTS2 and ADAMTS14, as has been reported for ADAMTS3 (6). The absence of ADAMTS2 or ADAMTS14 does not involve massive edema in our mouse models, suggesting that mutations in these genes should not be responsible for primary lymphedema. However, some reduction of their activity could be part of the numerous factors increasing the sensitivity to secondary lymphedema occurring after diverse types of injuries or trauma. This hypothesis is supported by our data showing that neolymphangiogenesis observed in healing cornea is almost completely prevented in absence of both ADAMTS2 and ADAMTS14. Efforts are being made now to develop inhibitors for specific ADAMTS, notably, for ADAMTS5, in order to find a medication able to limit cartilage degradation (25). Similar strategies could be used for ADAMTS2, ADAMTS3, and ADAMTS14 by focusing on their identical catalytic domain or on their ancillary domains, allowing their interactions with their substrates. If such inhibitors are obtained, they could help to fight conditions in which excessive or abnormal lymphangiogenesis is part of the pathological mechanisms, such as, for example, tumor aggressiveness and the formation of premetastatic niches in the lymph nodes.

## Methods

### Transgenic mice.

WT, *Adamts14^–/–^*, *Adamts2^–/–^*, and *Adamts2^–/–^Adamts14^–/–^* C57BL/6 mice were used for this study, either at postnatal day 6 or at 8 to 10 weeks of age (for descriptions, see refs. 15, 16). The animals were maintained on a 12-hour-light/dark cycle with free access to food and water.

### RT-PCR.

The different tissues were dissected and snap frozen. RNA was isolated using the Nucleospin RNA/protein extraction kit (Macherey Nagel, 740933.50). RT-PCR amplifications were performed using Tth DNA Polymerase (Roche). RT-PCR products were observed after electrophoresis in acrylamide gels and staining with Gel Star (Lonza, 50535). The following primers and cycles were used: 5′-CAGGCGCACACATAGTACCATCCA-3′ (reverse primer, sequence corresponding to exon 10) and 5′-CAGCCGCTACCTGCATTCCTATGA-3′ (forward primer, junction of exons 8 and 9) for *Adamts2* (28 cycles); 5′-GATACATCTCTGGGAGGCTGCTCCA-3′ (reverse primer, exon 22) and 5′-GCTGTGCCTATGTTGGTGACATCA-3′ (forward primer, junction of exons 20 and 21) for *Adamts3* (30 cycles); 5′-CCATCCTCGTGGTTGAGGGCACA-3′ (reverse primer, exon 7) and 5′-CTGATCATGGTGGGCTACCGACA-3′ (forward primer, exon 5) for *Adamts14* (30 cycles); and 5′-GATTCTGACTTAGAGGCGTTCAGT-3′ (reverse primer) and 5′-GTTCACCCACTAATAGGGAACGTGA-3′ (forward primer) for *28S* (internal control) (16 cycles). Due to the localizations of the target sequences of the primers, only a single product was amplified by qRT-PCR for each Adamts gene (at 257 bp for *Adamts2*, at 155 bp for *Adamts3,* and at 236 bp for *Adamts14*).

### Protein purification and VEGFC processing assay.

Recombinant ADAMTS2, ADAMTS3, and ADAMTS14 were purified as previously described (26). Stably transfected cells were grown in DMEM supplemented with 10% fetal calf serum. Upon confluence, growth medium was replaced by serum-free DMEM containing soybean trypsin inhibitor (40 μg/mL), heparin (5 μg/mL), and ZnCl_2_ (80 μM). After 48 hours, the medium was collected and the recombinant ADAMTS were purified through affinity chromatography (ConA-Sepharose [GE HealthCare, 17-0440-01] followed Heparin-Sepharose [GE HealthCare, 17-0467-01]). Note that this method leads to the purification of several ADAMTS polypeptides with different molecular weights (ref. 26 and Supplemental Figure 2) and different activities. In order to circumvent this problem, quantifications of recombinant ADAMTS were made after SDS-PAGE and staining with SYPRO Ruby using known concentrations of BSA as reference. Only the product around 100 kDa was taken into account, because this polypeptide corresponds to the expected molecular weight of the fully mature and most active form of ADAMTS2, ADAMTS3, and ADAMTS14. Concentrations of enzymes were adjusted around 10 ng per μL.

HEK293 cells (Invitrogen) expressing human pro-VEGFC were cultured for 48 hours in DMEM containing 0.1% FBS and 50 μM decanoyl-RVKR-CMK (furin inhibitor). For the processing assay, 20 μL conditioned medium was supplemented with NaCl (0.3 M final concentration) and 8 μL of either ADAMTS2, ADAMTS3, ADAMTS14, or control buffer, in the absence or presence of 25 mM EDTA (used as an inhibitor of metalloproteinases) in a total volume of 40 μL. After 18-hour incubation at 37°C, samples were denatured in reducing conditions (1× Laemmli sample buffer containing 200 mM DTT). Western blotting analyses were performed using a polyclonal goat anti-human VEGFC primary antibody (1:250, R&D Systems, AF752). Note that the VEGFC antibody does not react with the 29 kDa C-terminal domain of VEGFC and shows relatively low affinity for the fully processed mature 21 kDa form.

### Determination of the cleavage site in pro-VEGFC by ADAMTS2, ADAMTS3, and ADAMTS14.

HEK293 cells expressing human pro-VEGFC in a doxycycline-dependent manner were cultured for 60 hours in serum-free DMEM containing 1% nonessential amino acids (Biowest). The conditioned medium containing pro-VEGFC was collected with 0.1 mM AEBSF and concentrated on a 3 kDa cutoff filter (Amicon Ultra-15, PLBC, membrane Ultracel-PL, 3 kDa, MilliporeSigma). Total protein content was quantified (Micro BCA kit, Thermo Fisher Scientific) and 60 μg protein was incubated with control buffer or ADAMTS2, ADAMTS3, or ADAMTS14 for 18 hours at 37°C in 50 mM Tris-HCl, pH 8.0, containing 2 mM CaCl_2_ and 0.6 M NaCl.

Samples were denatured in 4 M guanidine HCl and labeled with different ITRAQ labels (iTRAQ Reagents Multiplex Kit, Sciex) according to the manufacturer’s instructions. Proteins were reduced with 1 mM TCEP and alkylated with 5 mM iodoacetamide. Ammonium bicarbonate buffer (pH 8) was added to reach 25 mM before methanol acetone precipitation. The protein pellets were dissolved in NaOH, and protein quantification was performed with Nanodrop. The different protein samples were precipitated using a 2D clean-up kit (GE Healthcare, 80648451) according to the manufacturer’s instructions. Samples were resolubilized in ammonium bicarbonate and digested using multienzymatic limited digestion, as previously described (27). LC-MS/SM analyses were performed on an Acquity M-Class UPLC (Waters) in line with a Q Exactive Plus (Thermo Fisher Scientific), in nanoelectrospray-positive ion mode. The trap column was a Symmetry C18 5 μm (180 μm × 20 mm), and the analytical column was a HSS T3 C18 1.8 μm (75 μm × 250 mm) (Waters Corp.). The samples were loaded at 20 μL/min on the trap column in 98% solvent A over 3 minutes and subsequently separated on the analytical column at a flow rate of 600 nL/min, with the following linear gradient: initial conditions, 98% A; 5 minutes, 93% A; 60 minutes, 70% A; 70 minutes, 60% A; and 73 minutes, 15% A, before maintenance for 5 minutes, after which the column was reconditioned in initial conditions. Solvent A is 0.1% formic acid in water, and solvent B is 0.1% formic acid in acetonitrile. The total run time was 60 minutes. A TopN-MSMS mass spectrometer method was used, where *N* was set to 15; this means that the spectrometer acquired 1 full MS spectrum, selected the 15 most intense peaks in this spectrum (singly charged and unassigned charge precursors excluded), and made a full MS2 spectrum of each of these 15 compounds. The parameters for MS spectrum acquisition were as follows: mass range from 400 to 2000 *m/z*; resolution, 70,000; and automatic gain control (AGC) target of 1 × 10^6^ or maximum injection time of 250 milliseconds. The parameters for MS2 spectrum acquisition were as follows: isolation window, 2.0 *m/z*; normalized collision energy, 30; resolution of 17,500; and AGC target of 2 × 10^5^ or maximum injection time of 120 milliseconds and a fixed first mass set at 100 *m/z*. The main parameters for QExactive Plus tune were as follows: spray voltage, 2.3 kV; capillary temperature, 270°C; and S-Lens RF level, 50.0. Data processing was performed by PEAKS X (BSI Bioinformatics Solutions). Relative quantitation was performed at the peptide level by considering the area under the curve of the corresponding extracted ion chromatograms.

### Activation of VEGFC pathway.

Primary human LECs (HMVEC-dLy, Lonza, CC-2810) were cultured in EGM2-MV medium in 6-well plate until confluence. HEK293 cells expressing human pro-VEGFC were cultured for 48 hours in DMEM containing 0.1% FBS. Conditioned medium (70 μL) was supplemented with NaCl (0.3 M final concentration) and 28 μL ADAMTS2, ADAMTS3, ADAMTS14, or control buffer in the absence or presence of 25 mM EDTA (used as an inhibitor of metalloproteinases) in a total volume of 140 μL. After a 18-hour incubation at 37°C, 20 μL or 100 μL of the incubation mixes were added to 1 mL serum-free EBM-2 medium (CC-3156, Lonza). LEC culture media were then replaced by the different digestion mixes. After 5 minutes, LECs were then lysed with cell lysis buffer 1× (9803, Cell Signaling) containing phosphatase and protease inhibitors (Complete and phosSTOP, Roche). Equivalent amounts of proteins were separated on polyacrylamide gels (7.5%) and transferred onto PVDF membranes. Membranes were then blocked, incubated overnight at 4°C with a pVEGFR3 (CY1115, Cell Applications) or a pVEGFR2-specific antibody (Tyr1175, no. 2478, Cell Signaling), washed extensively, and incubated for 1 hour at room temperature with horseradish peroxidase–coupled secondary antibody (P0217, Dako). After washing, the peroxidase activity was revealed with an enhanced chemiluminescence assay (ECL Prime Western Blotting System, MilliporeSigma) in an ImageQuant LAS 4000 (GE Healthcare). The membranes were then stripped, incubated with antibodies against total VEGFR3 (MAB3757 Merck Millipore) or total VEGFR2 (no. 2479 Cell Signaling), respectively, and revealed as described above.

### Hind limb and tail thickness measurements.

Images of hind limb and tail base from 8-week-old mice were acquired with a camera-equipped dissection microscope (Optika). The hind limb thickness was measured using the ImageJ software (NIH), just to the front of the first footpad, and the tail diameter was measured at the tail base.

### Wet-to-dry weight ratio.

Tail skins were removed and weighed (wet weight), dried for 72 hours in an oven at 60°C, and weighed again (dry weight). These measurements were used to determine the water content in the skin.

### Immunohistological analyses.

Tissues were fixed in a 4 % paraformaldehyde solution (and eventually decalcified in 0.5 m EDTA, pH 8, for 3 weeks in the case of hind limb and tail base samples) and paraffin embedded. Tissue sections (5 μm) were deparaffined, rehydrated, and stained with hematoxylin and eosin for general histological examination. The percentage of dermis area in hind limb and tail was quantified using ImageJ software as a percentage of the total tissue area.

For immunofluorescence, sections were treated (antigen retrieval, with Target retrieval solution, Dako) and then blocked with Universal blocking Reagent (HK085-5K BioGenex). Goat anti-LYVE1 antibody (1:100, AF2125, R&D Systems), and/or rat anti-CD31 antibody (1:150, DIA-310, Dianova), and/or rat anti-F4/80 antibody (1:100, Ab16911, Abcam) were incubated overnight at 4°C. After washes with PBS, Alexa Fluor 488–coupled rabbit anti-goat antibody (1:200; A21222, Invitrogen) was incubated at room temperature for 1 hour. After washes with PBS, Alexa Fluor 546–coupled goat anti-rat antibody (1:200; A11081, Invitrogen) was incubated at room temperature for 1 hour. Slides were then washed with PBS and mounted using Dako fluorescent mounting medium. Slides were scanned using a Nanozoomer 2.0 scanner (Hamamatsu) and visualized using NDPviewer (Hamamatsu). Dermal lymphatic count and dermal blood vessels per surface area were quantified using ImageJ software.

### Staining of lymphatic vessels on whole-mount ear skins from adult mice.

Ears from 8- to 10-week-old mice were collected and fixed in 4% paraformaldehyde overnight at 4°C. For each ear, the dorsal skin was separated from the rest of the tissue (central cartilage and the adhering ventral skin). Whole dorsal ear skins were fixed in methanol for 1 hour at –20°C and then blocked with 3% milk and 0.2% Triton X-100 for 1 hour at room temperature. They were then incubated with a polyclonal goat anti-LYVE1 antibody (1:200; AF2125, R&D Systems) and rat anti-CD31 antibody (1:150, DIA-310, Dianova) overnight at room temperature. After washing in PBS, an Alexa Fluor 488–coupled rabbit anti-goat antibody (1:200; A21222, Invitrogen) was added for 2 hours at room temperature. After washes with PBS, Alexa Fluor 546–coupled goat anti rat antibody (1:200; A11081, Invitrogen) was incubated at room temperature for 2 hours. Slides were then washed with PBS and mounted using Dako fluorescent mounting medium (Dako). Slides were scanned using a Nanozoomer 2.0 scanner (Hamamatsu) and visualized using NDPviewer (Hamamatsu). The lymphatic vessel frequency, density, and thickness were quantified using ImageJ software.

### Functional analysis of the lymphatic network in vivo.

To evaluate lymphatic drainage, 30 μL of a 3% solution of Evans blue dye (MilliporeSigma) in PBS was injected into the footpads of anesthetized mouse hind limbs. Thirty minutes after injection, mice were sacrificed and dissected to expose the inguinal lymph node. The images were acquired with a camera-equipped dissection microscope (Optika). The blue dye intensity was measured using ImageJ software. Results are expressed as a percentage of WT control.

### In vivo lymphangiogenesis.

Thermal cauterization was induced on the corneas of 8 to 10-week-old mice. After 7 days, corneas were dissected and whole mounted for immunostaining as previously described (28–30) or snap frozen for RNA extraction. To visualize lymphatic and blood vessels, corneas were fixed in methanol at –20°C for 1 hour and then blocked with 3% milk for 1 hour at room temperature. Corneas were then incubated overnight at room temperature with a polyclonal goat anti-mouse LYVE1 antibody (1:200; AF2125, R&D Systems) and with a monoclonal rat anti-mouse CD31 (1:200; 553370, BD Biosciences). After washing in PBS, an Alexa Fluor 488–coupled rabbit anti-goat antibody (1:200; A21222, Invitrogen) was added for 2 hours at room temperature. After washing in PBS, an Alexa Fluor 546–coupled goat anti-rat antibody (1:200; A-11081, Thermo Fisher Scientific) was added for 2 hours at room temperature. After washing, the slides were scanned using a Nanozoomer 2.0 scanner (Hamamatsu) and visualized using NDPviewer (Hamamatsu). The lymphangiogenic responses were analyzed using a described computerized method (28, 31). All the results were normalized to the total cornea area and are expressed as a percentage of WT control.

### Statistics.

Results were illustrated with GraphPad Prism 8.0 and were expressed as medians with interquartile ranges for different experiments. Statistical analyses were carried out with SigmaPlot 14.0 software using the nonparametric Kruskal-Wallis test to test whether measurements performed on the 4 genotypes of mice have the same distribution. When tests revealed differences, Holm-Šidák post hoc test was conducted to determine which groups were different from others. The Kolmogorov-Smirnov test was used to compare the shapes of the distribution curves, and the significance levels were adjusted with Bonferroni’s correction for multiple comparisons. *P* values of less than 0.05 were considered significant. The experiments were not randomized. Analysis and quantifications were not blinded.

### Study approval.

All animal experiments were conducted at the GIGA Animal Facility of the University of Liège in accordance with the Federation of European Laboratory Animal Science Associations and after approval from the local ethical committee at the University of Liège (approval no. 1964 and 2151).

## Author contributions

LD designed, performed, and analyzed experiments and wrote the manuscript. LJ contributed to the pro-VEGFC activation analyses and to the determination of the cleavage site in pro-VEGFC by ADAMTS2, ADAMTS3, and ADAMTS14. FM designed, performed, and analyzed experiments. SB performed all computerized quantifications. CM contributed to immunostaining and mouse breeding. CFD participated in experimental design and data interpretation. AN funded and supervised the project. ACMAC supervised, funded, and designed the project; interpreted the data; and wrote the manuscript.

## Supplementary Material

Supplemental data

## Version 1 03/22/2022

In-Press Preview

## Version 2 04/22/2022

Electronic publication
