# Supplementary material for: ADAMTS2 and ADAMTS14 can substitute for ADAMTS3 in adults for pro-VEGFC activation and lymphatic homeostasis
Source: JCI Insight. 2022 Apr 22;7(8):e151509. doi: 10.1172/jci.insight.151509 (PMC9089798; doi:10.1172/jci.insight.151509)
Supplement: Supplemental data [file jciinsight-7-151509-s143.pdf]

ADAMTS2 and ADAMTS14 substitute ADAMTS3 in adults for proVEGFC activation and lymphatic homeostasis.

Laura Dupont[1, 2], Loïc Joannes[2], Florent Morfoisse[1, 3], Silvia Blacher[1], Christine Monseur[2], Christophe F. Deroanne[2], Agnès Noël[1]*, Alain CMA Colige[2]*

[1]Laboratory of Tumor and Developmental Biology, GIGA-R, University of Liege, 4000 Sart Tilman, Belgium.
[2]Laboratory of Connective Tissues Biology, GIGA-R, University of Liege, 4000 Sart Tilman, Belgium.
[3]Institute of Metabolic and Cardiovascular diseases of Toulouse, INSERM UMR 1048, 31432 Toulouse, France.
*These authors contributed equally.

Corresponding author: Dupont Laura
Phone Number: +32/43669293
Address : Avenue Hippocrate n°13 B23/+3, 4000 Sart Tilman, Belgium.
E-mail address : ldupont@uliege.be

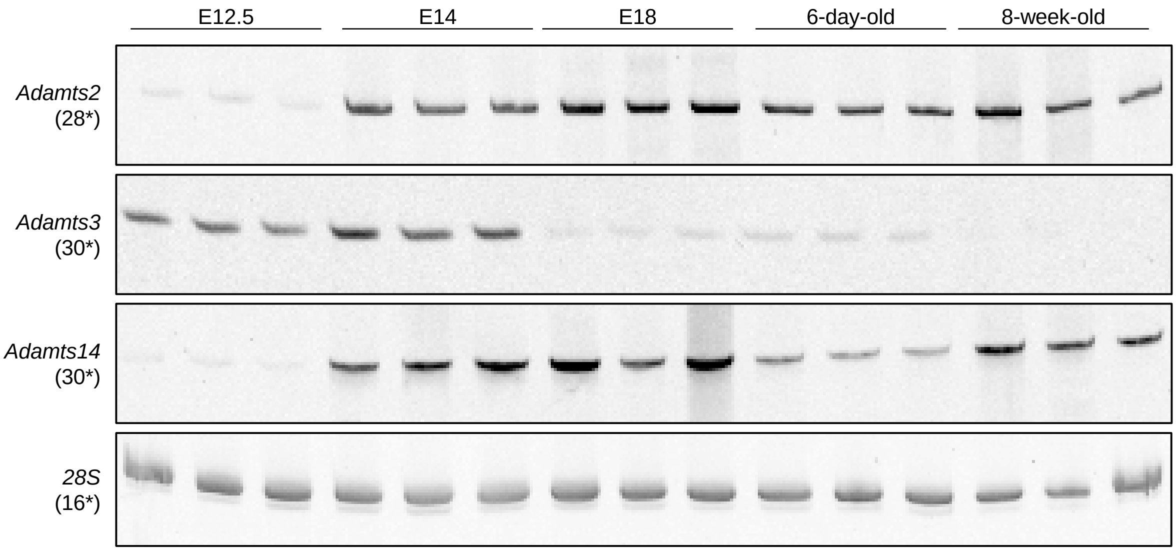

<u>Figure S1</u>: RT-PCR evaluation of the relative expressions of *Adamts2*, *Adamts3* and *Adamts14* at different developmental stages.

Total RNA was purified from embryos (whole body without the head and internal organs) at 12.5 and 14 days post coitus (E12.5 and E14) and from skin of embryos at E18 and of 6-day-old and 8-week-old mice. n = 3 at each timing.

Adamts3 is highly expressed at E12.5 and E14, but is strongly reduced at later stages. Conversely, the expression of *Adamts2* and *Adamts14* is very low at E12.5, then increases in late embryogenesis and remains significant at later stages. The size of the qRT-PCR products were 212 base pairs (bp) for *28S*, 257 bp for *Adamts2*, 155 bp for *Adamts3* and 236 bp for *Adamts14*. The numbers of PCR cycles are indicated (*).

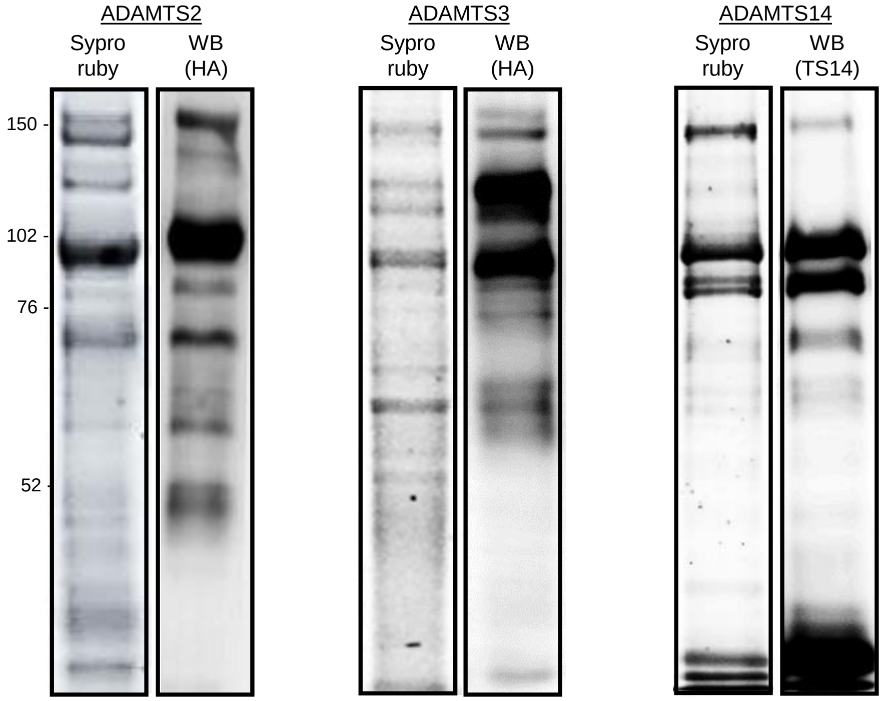

<u>Figure S2</u>: Characterizations of the fractions containing active recombinant ADAMTS2, ADAMTS3 or ADAMTS14. Recombinant ADAMTS2, ADAMTS3 and ADAMTS14 were purified as previously described (26). Stably transfected HEK-293 cells were grown in DMEM supplemented with 10% fetal calf serum. Upon confluence, the growth medium was replaced by serum free DMEM containing soybean trypsin inhibitor (40 µg/ml), heparin (5 µg/ml), and ZnCl2 (80 µM). After 48 h, the medium was collected and the recombinant ADAMTS were purified through affinity chromatography (ConA-Sepharose followed Heparin-Sepharose). Note that this method, which is the most efficient described so far (*Challenges and Solutions for Purification of ADAMTS Proteases: An Overview. Colige A. Methods Mol Biol. 2020;2043:45-53*), leads to the purification of several ADAMTS polypeptides with different molecular weights and different activities (26). In order to circumvent this problem, quantifications of recombinant ADAMTS were made after SDS-PAGE (10 µl of samples) and staining with SYPRO Ruby using known concentrations of BSA as reference. For comparison purposes, Western blot analyses were also performed (5 µl of samples) using an antibody specific for HA-flag (for ADAMTS2 and ADAMTS3) or for ADAMTS14. Only the product around 100 kDa was taken into account, since this polypeptide corresponds to the expected molecular weight of the fully mature and most active form of ADAMTS2, 3 and 14.

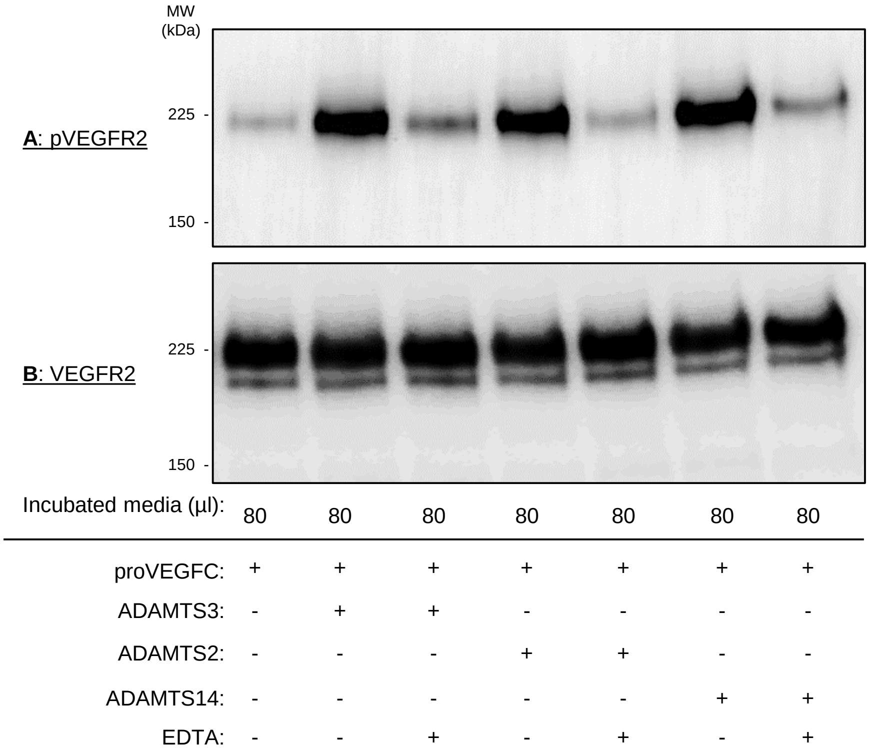

Figure S3: Phosphorylation of VEGFR2 by proVEGFC activated or not by ADAMTS3, ADAMTS2 or ADAMTS14.

Conditioned medium from HEK293 cells expressing full-length proVEGFC was first incubated for 18 hours with buffer alone (lane 1, negative control), ADAMTS3 (as positive control), ADAMTS2 or ADAMTS14, in the presence or absence of EDTA used as inhibitor. These different pretreated media were then added (80 µL) into 1 ml of serum-free EBM-2 on LECs cultures. After 5 min, cells were lysed, and phosphorylated VEGFR2 (A: pVEGFR2) was visualized by western blotting. After stripping of the antibodies, the same membrane was then used to visualize total VEGFR2 (B). Treatment of the proVEGFC-rich conditioned medium with active ADAMTS3, ADAMTS2 and ADAMTS14 stimulates the phosphorylation of VEGFR2, while the total amount of VEGFR2 remained unchanged, demonstrating that the processing of proVEGFC by ADAMTS2, ADAMTS3 or ADAMTS14 similarly leads to its activation.

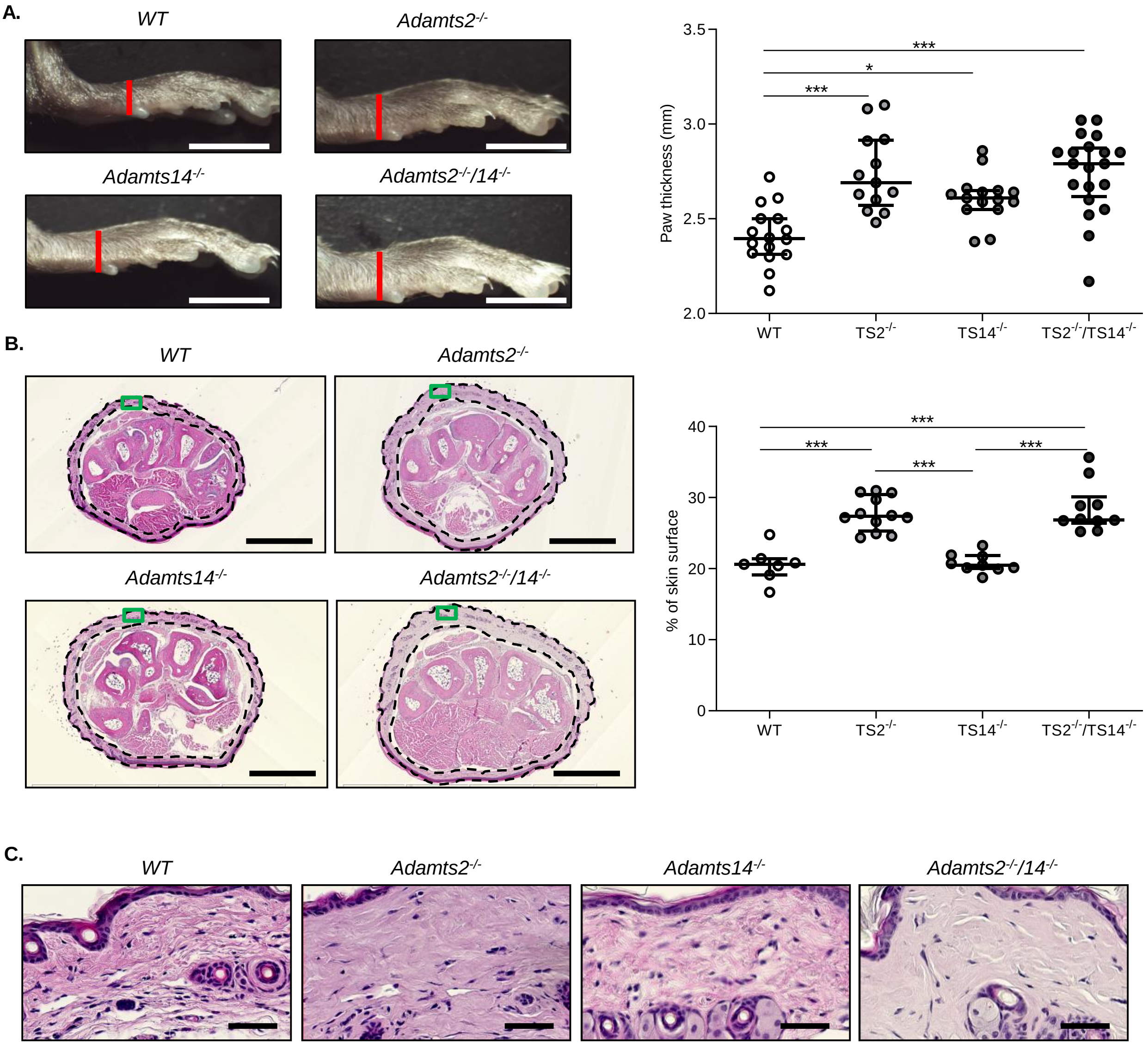

<u>Figure S4:</u> Skin swelling in absence of *Adamts2*.

A. Representative pictures of hind limbs of 8-week-old mice (scale bar: 5mm). Potential skin swelling was evaluated by measuring hind limb paw thickness immediately before the first footpad (red lines on pictures). As compared to WT mice, increased diameters were observed in $TS2^{-/-}$, $TS14^{-/-}$ and $TS2^{-/-}TS14^{-/-}$ mice. B. Hematoxylin-eosin staining on paraffin-embedded sections of hind limb (immediately before the first footpad) were performed to further characterize which tissue compartment is responsible for the increased diameter (scale bar: 1mm). For each sections of each genotype, the specific surfaces covered by the dermis (delimited by the black dotted lines) were determined (ImageJ software) and were found to be increased in $TS2^{-/-}$ and $TS2^{-/-}TS14^{-/-}$ mice. C. Higher magnification of pictures in B (corresponding to green boxes) showing a dermis less dense and swollen in $TS2^{-/-}$ and $TS2^{-/-}$ $TS14^{-/-}$ mice (scale bar: 50µm). Statistical analyses were performed using Kruskal-Wallis test followed by Holm-Sidak *post hoc* test for multiple comparisons. *$P < 0.05$; **$P < 0.01$; ***$P < 0.001$.

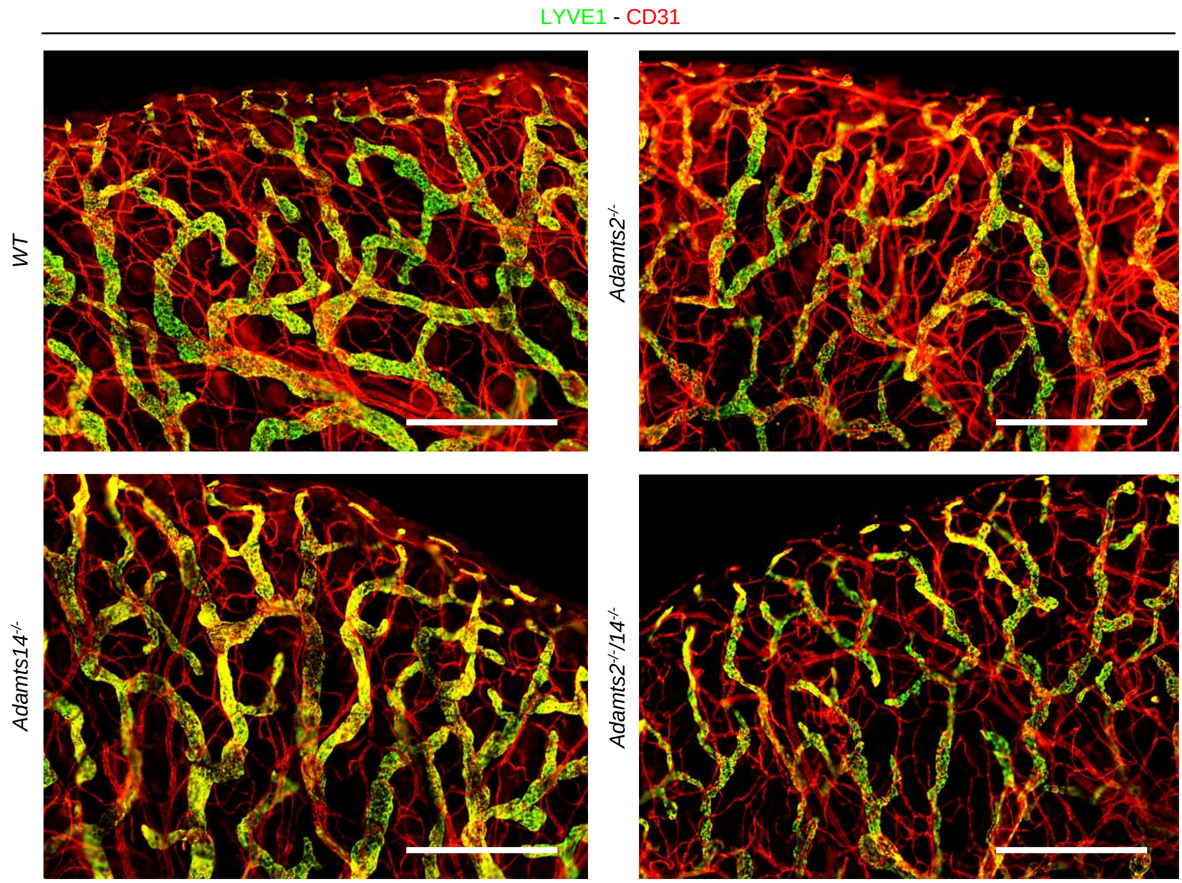

<u>Figure S5:</u> Absence of *Adamts2* and *Adamts14* does not affect the overall structure of the blood vessel network in ear skin.

LYVE1 (green) and CD31 (red) double immunofluorescence on whole-mount ear skin from 8-week-old WT, $TS2^{-/-}$, $TS14^{-/-}$ and $TS2^{-/-}TS14^{-/-}$ mice (Scale bar: 500µm). Lymphatics are mostly stained in yellow/orange because they express both LYVE1 and CD31. A similar and dense network of blood vessels (in red) was observed in the four genotypes.

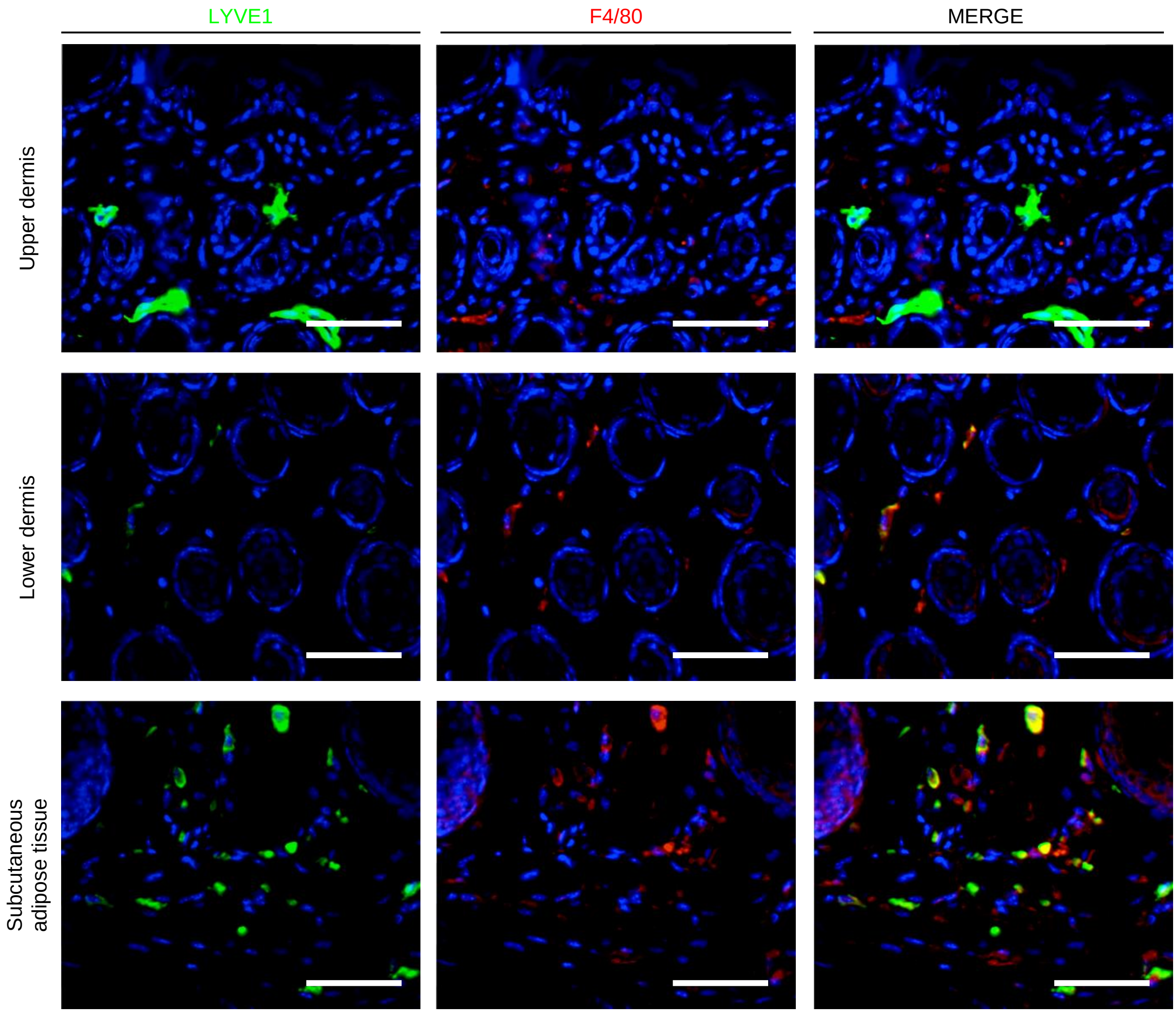

<u>Figure S6:</u> Lymphatic vessels are mainly localized in the upper dermis of skin in 6-day-old mice.

LYVE1 (lymphatic vessels and macrophages; green) and F4/80 (macrophages; red) double immunofluorescence on paraffin-embedded section of back skin from postnatal (P6) WT mice (scale bar: 50µm). Lymphatic vessels (green) are mainly present in the upper dermis, while most of the green cells identified in the lower dermis and in the subcutaneous adipose tissue are also positive for F4/80 which identifies them as macrophages.

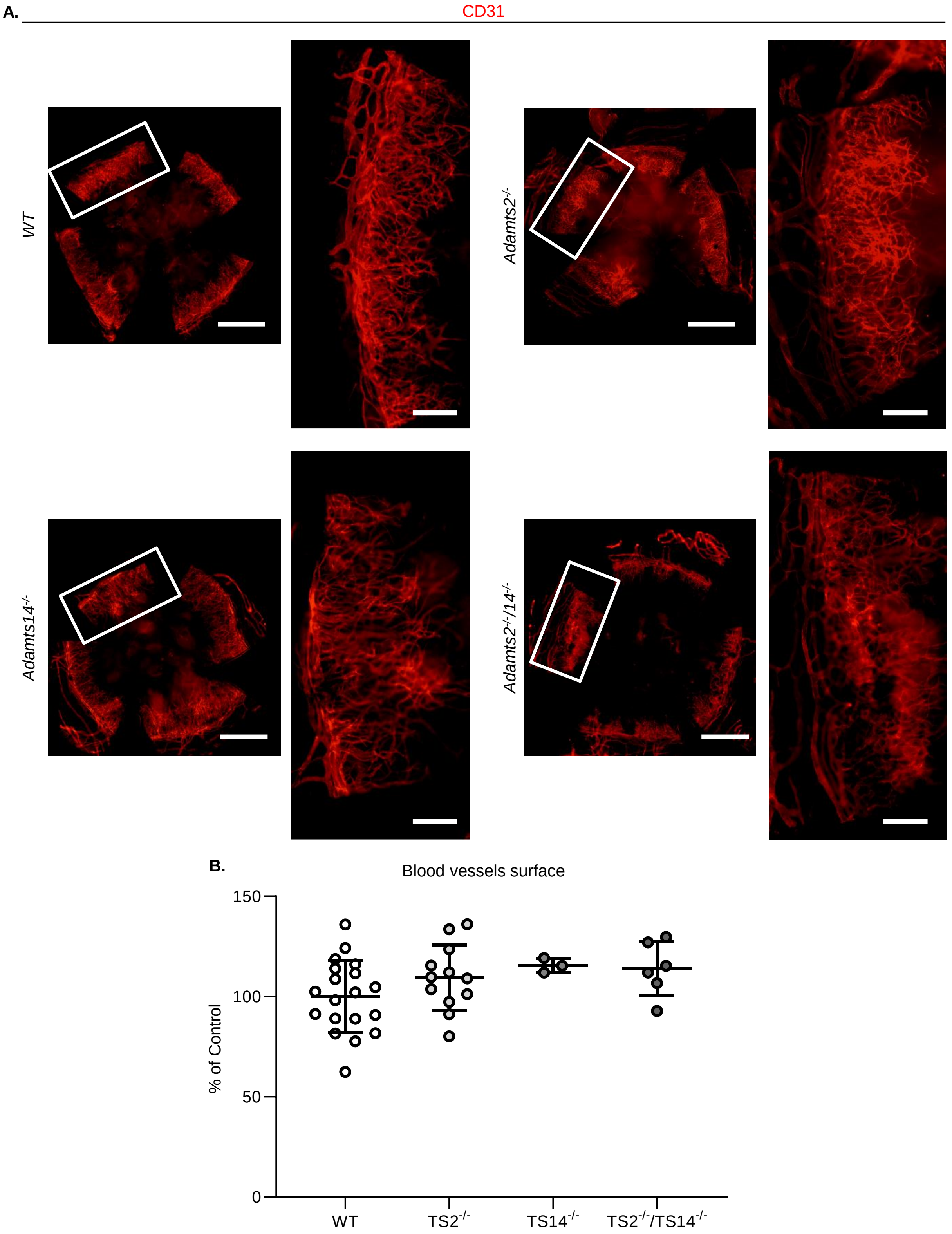

Figure S7: Angiogenesis resulting from LASER thermal cauterization of cornea is not affected in absence of *Adamts2* and/or *Adamts14*.

A. Blood vessels visualization using CD31 immunofluorescence on whole-mount cornea 7 days after thermal cauterization in WT, TS2$^{-/-}$, TS14$^{-/-}$ and TS2$^{-/-}$TS14$^{-/-}$ mice. The whole corneas (scale bar: 1mm), and an enlarged view of part of them (white box) (scale bar: 250µm), are provided for each genotypes. B. Computerized quantification of the blood vessels surface are expressed as percentage of the WT control. No statistical difference was observed between each genotypes. Statistical analyses were performed using Kruskal-Wallis test (p=0,165).
